# Evolutionary Rate Heterogeneity of Primary and Secondary Metabolic Pathway Genes in *Arabidopsis thaliana*

**DOI:** 10.1093/gbe/evv217

**Published:** 2015-11-10

**Authors:** Dola Mukherjee, Ashutosh Mukherjee, Tapash Chandra Ghosh

**Affiliations:** ^1^Bioinformatics Centre, Bose Institute, Kolkata, West Bengal, India; ^2^Department of Botany, Vivekananda College, Thakurpukur, Kolkata, West Bengal, India

**Keywords:** metabolic pathway genes, effective number of codons, multifunctionality, principal component analysis

## Abstract

Primary metabolism is essential to plants for growth and development, and secondary metabolism helps plants to interact with the environment. Many plant metabolites are industrially important. These metabolites are produced by plants through complex metabolic pathways. Lack of knowledge about these pathways is hindering the successful breeding practices for these metabolites. For a better knowledge of the metabolism in plants as a whole, evolutionary rate variation of primary and secondary metabolic pathway genes is a prerequisite. In this study, evolutionary rate variation of primary and secondary metabolic pathway genes has been analyzed in the model plant *Arabidopsis thaliana*. Primary metabolic pathway genes were found to be more conserved than secondary metabolic pathway genes. Several factors such as gene structure, expression level, tissue specificity, multifunctionality, and domain number are the key factors behind this evolutionary rate variation. This study will help to better understand the evolutionary dynamics of plant metabolism.

## Introduction

Being sessile organisms, plants tolerate constantly changing environments over their whole lifespan (Milo and Last 2012). To combat this, plants produce an enormous array of chemicals as unique adaptive strategies (Weng and Noel 2012; Weng 2014). These chemicals are synthesized or deconstructed by a collection of enzyme-catalyzed chemical reactions called metabolism (Weng and Noel 2012). A set of enzymes that catalyze sequential reactions in a highly concerned manner form the metabolic pathway (Weng 2014). Metabolism meets two apparently conflicting requirements: to maintain the homoeostasis necessary for a living organism and to respond dynamically to the constantly changing environment (Milo and Last 2012). Primary metabolic pathways required for the survival of plants are conserved in all living organisms (Mullins et al. 2008; Weng 2014), whereas secondary or specialized metabolic pathways have a multitude of roles in plant interaction with the environment (Zhao et al. 2013; Weng 2014). The majority of plant metabolites is secondary metabolites and has a direct effect on plant fitness (Zhao et al. 2013).

There are several differences in primary and secondary metabolic pathway enzymes. In primary metabolism, the demand for high metabolic flux forces a major selective pressure on the evolution of its enzymes (Nam et al. 2012). As a result, these enzymes show low levels of catalytic promiscuity (Bar-Even et al. 2011). On the other hand, for secondary metabolic pathway enzymes, the selection pressure is to optimize the regulation, control, and localization with the fluctuating environmental conditions rather than to increase the metabolic flux (Bar-Even et al. 2011; Weng and Noel 2012). Under such circumstances, efficiency and precision are traded for synthesis of a wider diversity of products to cope up with the spatially and temporally changing environments (Weng and Noel 2012). This makes the secondary metabolic pathway enzymes more promiscuous than primary metabolic pathway enzymes (Weng and Noel 2012). Moreover, specialized metabolic enzymes are approximately 30 times less active than primary metabolic enzymes (Bar-Even et al. 2011). Evolutionary selection pressures as well as physicochemical constrains affect enzymes (Bar-Even et al. 2011), and functional promiscuity can potentiate adaptive evolution (DePristo 2007). As chemical diversity shapes biological diversity, the principles of evolution must be relevant to chemical diversity (Firn and Jones 2009). Thus, understanding the effect of selection on genes involved in pathways has received ample attention in the study of molecular evolution recently (Clotault et al. 2012). According to Weng (2014), metabolism offers an attractive platform to investigate evolutionary processes that lead to biological complexity. At present, the challenge in studying metabolism is to understand how evolution worked and shaped the characteristics of extant plants (Milo and Last 2012). Additionally, various plant metabolites are used for the production of dyes, medicines, flavors, insecticides, and fragrances (Verpoorte and Memelink 2002) and are thus of industrial, pharmaceutical, and agricultural interest (Zhao et al. 2013). Several primary metabolites such as starch, vitamins, and amino acids are potential candidates for metabolic engineering (Trethewey 2004). However, there is a scarcity in the comparative studies of primary and secondary metabolism (Weng and Noel 2012). Few studies have been conducted on the evolutionary rate variation of specific metabolic pathways in plants (Rausher et al. 2008; Ramsay et al. 2009) and thus involve a relatively small number of genes. Poor characterization of plant secondary metabolic pathways is a major constraint for successful molecular breeding practices (Verpoorte and Memelink 2002). Elucidation of plant metabolite biosynthesis will thus provide an expanded knowledge base and molecular tools for the genetic manipulation of biochemical pathways (Zhao et al. 2013).

It will also be of immense interest to study whether rapid enzyme evolution in plants is facilitated by other molecular machineries encoded by the plant genome (Weng 2014). Different attributes shape the evolutionary dynamics of a gene (Yang and Gaut 2011). It has been previously reported that the characteristics of gene such as gene length (Marais and Duret 2001) and intron number (Larracuente et al. 2008) correlate with both synonymous and nonsynonymous evolution. Other factors such as GC content, untranslated region (UTR) length, expression level, tissue specificity, and multifunctionality also correlate with the evolutionary rate of genes of *A. thaliana *(Yang and Gaut 2011). Protein domains are basic evolutionary units (Fong et al. 2007), and they are likely to have a highly conserved location within proteins (Pils et al. 2005). Effective number of codons (EN_c_) has also been showed to modulate evolutionary rates in *Drosophila* (Han et al. 2013). Domains typically cover a majority of a protein sequence and play a crucial role in protein evolution (Toll-Riera and Albà 2013).

Primary metabolic pathways are well established (Castillo et al. 2013). Although a significant portion of the plant genome is involved in specialized metabolic pathways (Castillo et al. 2013), genomic analysis generally fails (Bocobza et al. 2012) because the taxonomically narrowly distributed pathways lack true orthologs (Castillo et al. 2013). Complete genome sequences of the model plant *Arabidopsis thaliana *(L.) Heynh. (Arabidopsis Genome Initiative 2000) have helped to resolve many problems regarding gene regulation and functional compensation (Hanada et al. 2011). However, lack of studies regarding variation of evolutionary rate between primary and secondary metabolic pathway genes was due to the absence of a closely related genome that allows accurate ortholog identification (Gaut and Ross-Ibarra 2008). However, the availability of *A. lyrata* genome (Hu et al. 2011) made it possible to correctly identify the orthologs for *A. thaliana* (Yang and Gaut 2011). Additionally, these two species have diverged approximately 13 Ma (Beilstein et al. 2010) and have approximately 80% sequence identity over whole-genome alignments (Hu et al. 2011). Thus, the study of *A. thaliana *genes with the help of *A. lyrata* orthologs will give us an accurate measure of evolutionary rate variation in primary and secondary metabolic pathway genes. Previously, genome-wide patterns of evolutionary rate variation among *A. thaliana* nuclear genes and its correlates have been studied (Yang and Gaut 2011). However, primary and secondary metabolic pathway genes should show difference in evolutionary rates as they act under different selective pressures. Hence, we have analyzed the difference in evolutionary rates and the factors that shape this variation in *A. thaliana*. We have addressed three questions. First, what is the difference in evolutionary rates between primary and secondary metabolic pathway genes? Second, what are the correlates of the evolutionary rate? Third, what is the relative contribution of these correlates in the evolutionary rate variation? Our study suggests that primary metabolic pathway genes are more conserved than secondary metabolic pathway genes of *A. thaliana*. This variation is mainly governed by gene structure, expression level, tissue specificity, multifunctionality, and domain number. The differences in nonsynonymous substitutions in the two types of pathways are mainly due to factors related to gene expression, whereas the differences in synonymous substitutions are mainly due to gene-level variations. This information is valuable for further biotechnological studies.

## Materials and Methods

### Data Set Preparation and Evolutionary Rate Estimation

The KEGG Orthology for *A. thaliana* (ath00001.keg) was downloaded from KEGG database (Kanehisa and Goto 2000). We have chosen all the nuclear genes of primary and secondary metabolic pathways of *A. thaliana*. A list of all these pathways is given in supplementary material S1, Supplementary Material online. We have obtained a total of 2,030 genes for primary metabolism and 482 genes for secondary metabolism. We extracted the corresponding *Arabidopsis lyrata* orthologs (with 1:1 orthology and at least 80% sequence similarity) of *A. thaliana* genes from Ensembl Plants database (Kersey et al. 2014) using Biomart (Kinsella et al. 2011) as well as obtained their pairwise nonsynonymous (*d*_N_) and synonymous (*d*_S_) substitution rates to compute gene-specific evolutionary rate (*d*_N_/*d*_S_). These *d*_N_ and *d*_S_ values have been calculated using codeml from the PAML package (Yang 1997). Protein coding sequences of these genes were also acquired from Ensembl database. For genes with more than one isoform, the longest isoform was considered. The final data set comprised 2,030 primary metabolic and 273 secondary metabolic genes with available evolutionary rate for further analysis (supplementary material S2, Supplementary Material online).

### Determination of Potential Factors of Evolutionary Rates

Several factors such as gene structure, GC content, expression profile, multifunctionality, and protein domain organization have been analyzed to determine their potential to modulate the evolutionary rate. These are studied as follows.

### Gene Structure and GC Content

Gene, UTR, and coding DNA sequences (CDS) have been downloaded from Ensembl Plants database. A CDS that did not begin with an ATG start codon or did not end with the stop codon (TAG/TAA/TGA) or did not occur in multiples of three nucleotides has been discarded. We have calculated gene length, 5′- and 3′-UTR length, intron number, and average intron length as well as GC content of genes, 5′- and 3′-UTRs. GC_3_ has been calculated from CDS of the genes using CodonW (http://codonw.sourceforge.net/, last accessed June 15, 2015). To measure the state of codon usage bias of the genes, we have measured EN_c_ (Wright 1990) using CodonW. Pfam (Finn et al. 2014) domain annotations were obtained from Ensembl Biomart against the longest peptide and number of domains per gene was counted.

### Gene Expression Level and Pattern

The expression data were obtained from using Genevestigator (Hruz et al. 2008). Various microarray expression data of the *A. thaliana *(ATH1:22 k array) were obtained from Genevestigator plant biology version (https://www.genevestigator.com/gv/plant.jsp). The expression level of a gene was estimated by the average value of all the samples. The tissue specificity index τ was measured following Yanai et al. (2005) as follows:

$$\tau =\frac{\sum_{j}^{n}=1\left[1-\frac{\log S(i,j)}{\log S(i,\max)}\right]}{n-1}$$

where *n* is the number of tissues and conditions, and *S*(*i*, max) is the highest expression of gene *i* across the *n* tissues. The index τ ranges from 0 to 1, with a higher value signifying higher specificity. The index τ has been used because of its advantage over using expression breadth as reported previously (Liao and Zhang 2006). We have also collected Plant Ontology (PO) (Avraham et al. 2008) data for each gene to better understand the expression of a particular gene in different plant structures and developmental stages. The PO data have been obtained from Ensembl Plants (http://plants.ensembl.org/index.html).

### Function

According to Gene Ontology (GO) Slim annotations that classify proteins to obtain a high-level view of functions (Prachumwat and Li 2006), the multifunctionality of a gene has been assessed by counting the number of biological processes in which a gene takes part. The GO slim accessions were obtained from Ensembl Biomart (Kinsella et al. 2011).

### Statistical Analyses

Statistical analyses were performed using SPSS v.13. Mann–Whitney *U* test (Mann and Whitney 1947) was used to compare the average values of different variables between two classes of genes as the values were not normally distributed in our data set. For correlation analysis, we performed the Spearman’s rank correlation coefficient ρ (Spearman 1904), where the significant correlations were denoted by *P* < 0.05. For relative contribution analysis of each factor to evolutionary rate, a principal component analysis (PCA) was performed.

## Results and Discussion

### The Variation of Evolutionary Rates between Primary and Secondary Metabolic Pathway Genes

**Fig. 1.—:**
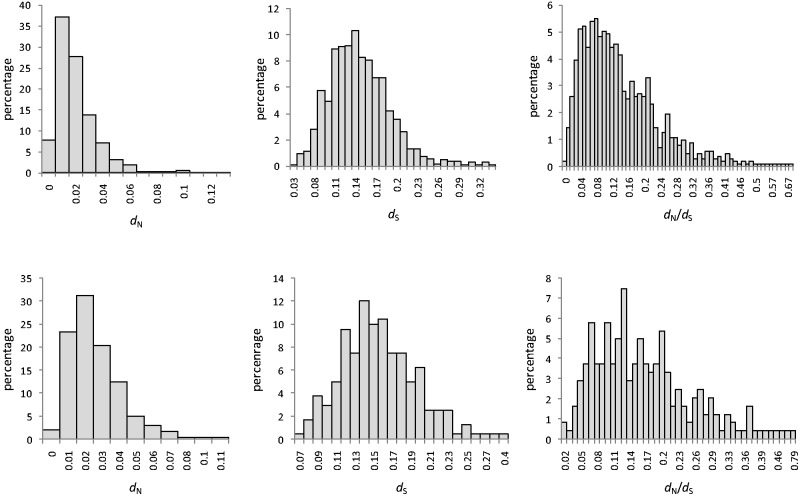
Distribution of *d*_N_, *d*_S_, and *d*_N_/*d*_S_ in primary (upper panel) and secondary (lower panel) metabolic pathway genes in *A. thaliana*.

This study clearly showed that primary metabolic pathway genes are more conserved than secondary metabolic pathway genes in *A. thaliana*. *d*_N_, *d*_S_, and *d*_N_/*d*_S_ were calculated for 1,035 primary and 241 secondary metabolic pathway genes. Frequency distributions of these three parameters are shown in figure 1. The average values of *d*_N_, *d*_S_, and *d*_N_/*d*_S_ were significantly (Mann–Whitney *U *test, *P* < 0.01 in all cases) different in primary and secondary metabolic pathway genes (table 1). The frequency distribution of *d*_N_, *d*_S_, and *d*_N_/*d*_S_ was also different in two types of pathways (fig. 1). Highest frequency (around 37%) of genes in primary metabolic pathways showed a *d*_N_ value of approximately 0.01, whereas the highest frequency (around 31%) of genes in secondary metabolic pathways showed a *d*_N_ value of approximately 0.02. Considering *d*_S_ values_, _around 10% of primary metabolic pathway genes showed a *d*_S_ value of approximately 0.14, whereas around 12% of secondary metabolic pathway genes showed a *d*_S_ value of approximately 0.14. Highest frequency (around 5.5%) of genes in primary metabolic pathways showed a *d*_N_/*d*_S_ value of approximately 0.08, whereas a highest frequency (around 7.46%) of genes in secondary metabolic pathways showed a *d*_N_/*d*_S_ value of approximately 0.13. The synonymous (*d*_N_) and nonsynonymous (*d*_S_) substitution rates were 1.3 and 1.06 times greater in secondary metabolic pathway genes, respectively. We also found that no gene in our data set showed *d*_N_/*d*_S_ value >1, which indicate that, on average, genes involved in primary and secondary metabolic pathways in *A. thaliana* are not under positive selection. In general, genes under positive selection are rare in *A. thaliana *genome (Yang and Gaut 2011). It is also noteworthy that *d*_N_ and *d*_S_ values were highly positively correlated (Spearman’s rank correlation ρ = 0.246 × 10^−^^6^). It suggests the effect of common evolutionary mechanisms on both synonymous and nonsynonymous sites of which at least one is shared mutation rates (Yang and Gaut 2011). The positive correlation of synonymous and nonsynonymous substitutions has also been found in *Drosophila* (Comeron and Kreitman 1998), indicating that synonymous substitutions are not independent of selective constraints acting on the amino acid level (Dunn et al. 2001).

**Table 1:** Evolutionary Rates for Primary and Secondary Metabolic Pathway Genes (*P* Values Were Obtained by Mann–Whitney *U *Test)

	Primary	Secondary	*P* value
*d*_N_			
Mean (SD)	0.02 (0.016)	0.026 (0.016)	1.73 × 10^−10^
CV	0.789	0.629	
Range	0.0008–0.135	0.002–0.111	
*d*_S_			
Mean (SD)	0.147 (0.044)	0.157 (0.043)	2.5 × 10^−4^
CV	0.305	0.276	
Range	0.031–0.371	0.072–0.399	
*d*_N_/*d*_S_			
Mean (SD)	0.142 (0.103)	0.170 (0.102)	1.19 × 10^−6^
CV	0.725	0.599	
Range	0.003–0.855	0.018–0.791	

The higher rate of evolution confers an advantage to genes involved in secondary metabolism. As the enzymes of secondary metabolism exhibit high plasticity (Khersonsky et al. 2006), a few mutations can increase the promiscuous activity. It has been shown that 10^4^- to 10^6^-fold improvement in enzyme plasticity has been achieved in response to a single mutation (Khersonsky et al. 2006), and this functional promiscuity may potentiate adaptive evolution (DePristo 2007). As primary metabolic pathway enzymes are directly involved in the core functioning of a plant, they show vanishingly low levels of promiscuity (Weng and Noel 2012). This explains that higher evolutionary rate of the genes involved in secondary metabolism gives the plant a selective advantage in the ever changing environment, whereas conserved nature of primary metabolic pathway genes assures the integrity of the core functioning. This finding also has implications in biotechnology, especially protein engineering. Enzyme-catalyzed industrial processes are increasing in various fields ranging from food processing to produce small molecule pharmaceuticals (Gustafsson et al. 2012). Our results suggest that, in case of secondary metabolic pathway genes of *A*. *thaliana*, protein engineering can result in the formation of new and novel metabolites that may be advantageous for the plant (as they accumulate more synonymous and nonsynonymous substitutions as well as more promiscuous than primary metabolic pathway genes). Indeed, several carotenoid biosynthetic enzymes such as synthases, desaturases, cyclases, and oxygenases have been altered for both substrate specificity and reaction selectivity by single amino acid substitutions to produce a plethora of novel carotenoids (Umeno et al. 2005; Tracewell and Arnold 2009). On the other hand, altering the highly conserved primary metabolic pathway genes can disrupt the normal functioning of the enzyme. Chen et al. (2010) showed that in a α-amylase (an enzyme from primary metabolic pathway) from *Bacillus* sp. strain TS-23, a mutation of Asp-234 and Asp-236 of conserved sequence region V (CSR-V) resulted in 33% and 86% reduction in specific activity (Chen et al. 2010). However, in another α-amylase from *Anoxybacillus* species, replacement of Ala-161 of CSR-V with an aspartic acid increased the specific activity (Ranjani et al. 2014). Thus, it is advisable that creating any mutation in primary metabolic genes should be performed with great care to retain the functionality of the protein.

### Effect of Different Factors on the Evolutionary Rate Variation among the Primary and Secondary Metabolic Pathway Genes in *A. thaliana*

One of the most important objectives of molecular evolution studies is to understand the factors that influence genetic variation in the genome (Clotault et al. 2012). Effect of mutation and selection has different effect on synonymous and nonsynonymous substitutions (Yang and Nielsen 1998). Although the occurrence of a synonymous mutation is assumed to have no effect on the fitness of the individual (Zhou et al. 2012), selection on synonymous sites has been shown to be associated with mRNA secondary structure and stability (Duan et al. 2003; Chamary and Hurst 2005; Stoletzki 2008; Gu et al. 2010) as well as protein expression (Zhou et al. 2010). Moreover, several gene properties can affect the mutation rate or the local selection environment, both of which encompass protein evolutionary rates (Chang and Liao 2013). If the influence on protein evolutionary rates was at the selection level, its correlation to *d*_N_ and *d*_N_/*d*_S_ should not differ considerably (Chang and Liao 2013). Considering all the above factors, we have measured the effect of several factors on *d*_N_, *d*_S_, and *d*_N_/*d*_S_. Many factors such as gene length (Stoletzki and Eyre-Walker 2007), gene compactness (Liao et al. 2006; Chang and Liao 2013), GC content (Ratnakumar et al. 2010), codon usage bias (Sharp and Li 1987; Urrutia and Hurst 2001; Yang and Gaut 2011), expression level (Pál et al. 2001; Subramanian and Kumar 2004; Rocha 2006), and tissue specificity (Larracuente et al. 2008; Slotte et al. 2011) have been shown to influence evolutionary rate. However, the relative importance of determinants for protein evolutionary rates varies widely among various taxa. For example, in yeasts, predominant factor determining the rate of protein evolution was found to be mRNA abundance (Drummond et al. 2006), whereas in mammals, gene compactness was found to have a stronger influence on protein evolutionary rates compared with the abundance of mRNA (Liao et al. 2006, 2010). Besides, coding sequence length showed the strongest positive correlation with protein evolutionary rates in flagellated algae (Chang and Liao 2013). We have thus focused on various parameters that could have affected the evolutionary rate variation in genes of primary and secondary metabolic pathways in *A*. *thaliana*.

### Effect of Gene Length and Gene Compactness

Gene length has been previously shown to have a negative correlation with evolutionary rate in *A*. *thaliana* (Yang and Gaut 2011). In this study, the average length of genes in the primary metabolic pathways (2,762.75 bp, *N* = 1,035) is significantly (Mann–Whitney *U *test, *P* = 3.1 × 10^−^^4^) higher than secondary metabolic pathway genes (2,439.63 bp, *N* = 241). We have found a significant negative correlation between gene length and *d*_N_, *d*_S_, and *d*_N_/*d*_S_ (table 2). Previously, Slotte et al. (2011) reported no correlation between *d*_N_/*d*_S_ and gene length when analyzing all genes in the *A. thaliana *genome. However, Yang and Gaut (2011) reported a strong negative correlation between gene length and *d*_N_, *d*_S_, and *d*_N_/*d*_S_. We have also studied the effect of different attributes of gene compactness, such as UTR length, intron length, and intron number on the evolutionary rate of primary and secondary metabolic pathway genes. Average length of 5′-UTR in the primary metabolic pathways (128.60 bp, *N* = 1,035) is significantly (Mann–Whitney *U *test, *P* = 8.9 × 10^−^^8^) higher than secondary metabolic pathway genes (96.49 bp, *N* = 241). Average length of 3′-UTR in the primary metabolic pathways (231.06 bp, *N* = 1,035) is significantly (Mann–Whitney *U *test, *P* = 7.5 × 10 ^−^^ 6^) higher than secondary metabolic pathway genes (192.87 bp, *N* = 241). Both 5′- and 3′-UTR lengths were found to be negatively significantly correlated with *d*_N_, *d*_S_, and *d*_N_/*d*_S_ (Spearman’s rank correlation, *P* < 0.001) (table 2). This is in accordance with the results obtained for all genes in *A*. *thaliana* (Yang and Gaut 2011). Our results showed that UTR length plays a significant role in the evolutionary rate variation in *A*. *thaliana*.

**Table 2:** Spearman’s Rank Correlations of Evolutionary Rates with Potentially Contributing Factors

Variables	Spearman’s ρ (*P* value)					
	*d*_N_		*d*_S_		*d*_N_/*d*_S_	
Gene structure and compactness						
Gene length	−0.170 (1.0 × 10^−6^)***		−0.199 (1.0 × 10^−6^)***		−0.96 (9.9 × 10^−4^)***	
Intron number	−0.08 (4.3 × 10^−3^)**		−0.248 (1.0 × 10^−6^)***		0.011 (NS)	
5′-UTR length	−0.277 (1.0 × 10^−6^)***		−0.176 (1.0 × 10^−6^)***		−0.214 (1.0 × 10^−6^)***	
3′-UTR length	−0.326 (1.0 × 10^−6^)***		−0.158 (1.0 × 10^−6^)***		−0.277 (1.0 × 10^−6^)***	
GC content						
5′-UTR	−0.170 (1.0 × 10^−6^)***		−0.167 (1.0 × 10^−6^)***		−0.103 (4.1 × 10^−4^)***	
3′-UTR	−0.204 (1.0 × 10^−6^)***		−0.145 (1.0 × 10^−6^)***		−0.151 (1.0 × 10^−6^)***	
GC_3_	−0.092 (1.6 × 10^−3^)**		0.05 (NS)		−0.111 (1.4 × 10^−4^)***	
Domain number	−0.1 (6.1 × 10^−4^)***		−0.073 (1.2 × 10^−2^)*		−0. 073 (1.2 × 10^−2^)*	
Expression						
Expression level	−0.461 (1.0 × 10^−6^)***		−0.151 (1.0 × 10^−6^)***		−0.405 (1.0 × 10^−6^)***	
Tissue specificity	0.241 (1.0 × 10^−6^)***		0.198 (1.0 × 10^−6^)***		0.170 (1.0 × 10^−6^)***	
ENc	0.151 (1.0 × 10^−6^)***		−0.031 (NS)		0.168 (1.0 × 10^−6^)***	
PO	−0.392 (1.0 × 10^−6^)***		−0.163 (1.0 × 10^−6^)***		−0.338 (1.0 × 10^−6^)***	
Multifunctionality						
GO slim	−0.359 (1.0 × 10^−6^)***		−0.142 (1.09 × 10^−6^)***		−0.308 (1.0 × 10^−6^)***	

Note.—NS, not significant. * *P*<0.05, ** *P* <0.01, *** *P* < 0.001.

**Fig. 2.—:**
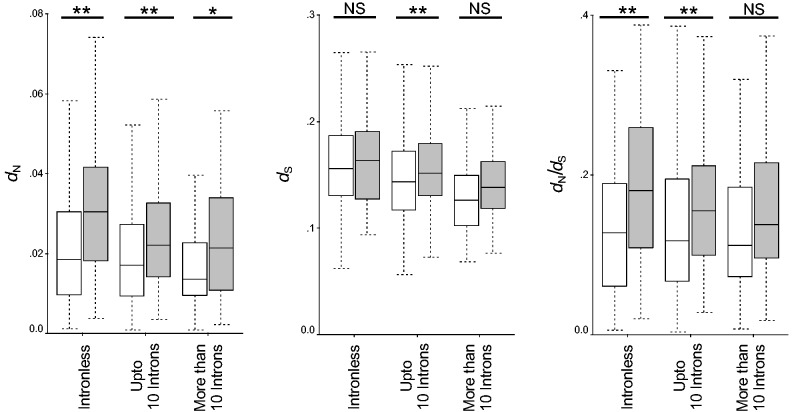
Boxplots showing distribution of *d*_N_, *d*_S_, and *d*_N_/*d*_S_ in primary (white boxes) and secondary (gray boxes) metabolic pathway genes in three groups according to the number of introns in *A. thaliana*. NS, not significant, * *P*<0.05, ** *P*<0.01.

**Fig. 3.—:**
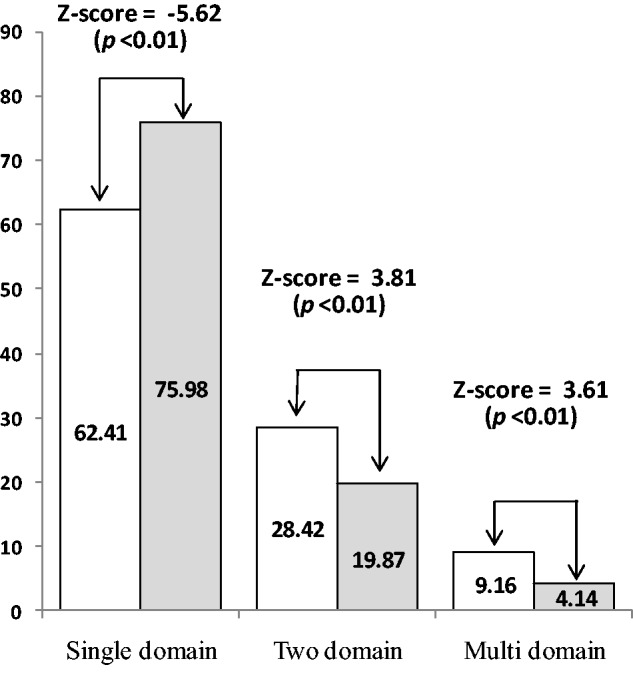
The bar diagram depicts the percentage of single domain, two domain, and multidomain proteins within primary and secondary metabolic pathway genes in *A. thaliana*. In each group, the white bar represents primary metabolic pathway genes whereas the gray bar belongs to secondary metabolic pathway genes.

Average intron length was not found to be significantly different in these two types of pathways (Mann–Whitney *U *test, *P* > 0.05). However, intron number was found to be significantly different between the two types of pathways (6.17, *N* = 976 and 4.30, *N* = 222, respectively, for primary and secondary metabolic pathways, Mann–Whitney *U *test, *P* = 1.86 × 10^−^^6^). Intron number was significantly correlated with *d*_N_ and *d*_S_, but not with *d*_N_/*d*_S_ (table 2). Thus, it was found that primary metabolic pathway genes were longer and less compact than secondary metabolic pathway genes, and these features are responsible for their evolutionary rate heterogeneity in *A. thaliana*. Earlier reports showed that shorter and intron-poor genes have either more variable (Jeffares et al. 2008; Lawniczak et al. 2008) or stronger (Castillo-Davis et al. 2002; Chiaromonte et al. 2003; Ren et al. 2006) expression levels. Regulatory responses can be delayed by introns and are selected against genes whose transcripts require rapid adjustment for survival of environmental challenges (Jeffares et al. 2008). Indeed, secondary metabolic genes that are generally expressed in response of environmental challenges are shorter as determined in our study. It is noteworthy that correlation of intron number was higher with *d*_S_ than *d*_N_. To better understand this variation, we have divided the data set into three groups: Intronless genes, genes with upto ten introns, and genes with more than ten introns. We have found no significant difference in *d*_S_ between intronless genes of primary and secondary metabolic pathway genes (fig. 2). This was also found in genes with more than ten introns. However, *d*_N_ was found to be significantly different in all three groups. However, when intron number increases to more than 10, the rate of synonymous substitutions became similar. This later observation is unclear at the moment. However, one reason behind this may be the very small number of secondary metabolic genes with more than ten introns (only 26), which probably gave a biased result here. However, in each group, nonsynonymous substitutions were significantly higher in primary than secondary metabolic genes. All these results show that introns significantly accumulate more synonymous substitutions in primary than secondary metabolic genes. Primary metabolic pathway genes contain significantly higher domain number (1.53 per gene, *N* = 1,034; Mann–Whitney *U *test, *P* = −5.51 × 10^−^^12^) than secondary metabolic pathway genes (1.26, *N* = 240). We have also analyzed the proportion of single, double, and multidomain proteins in the two types of pathways. In total, 62.41% and 75.98% of all proteins were single domain proteins in primary and secondary metabolic pathways, respectively, and they are significantly different (*Z* score = −5.62, *P* < 0.01). However, primary metabolic pathways significantly contain more number of double and multidomain proteins (fig. 3). There is a strong negative correlation between domain number per gene and evolutionary rates (table 2). Domain number was found to show higher correlation coefficient with *d*_N _than *d*_S_.

### Effect of GC Content Variation

GC content of the genes in the two types of pathways was not significantly different (Mann–Whitney *U *test, *P* = 0.675). However, GC content of UTRs was significantly different. Average GC content of 5′-UTR in the primary metabolic pathways (34.27%, *N* = 1,035) is significantly (Mann–Whitney *U *test, *P* = 9.3 × 10^−^^9^) higher than that in the secondary metabolic pathway genes (31.00%, *N* = 241). Average GC content of 3′-UTR in the primary metabolic pathways (29.58 bp, *N* = 1,035) is significantly (Mann–Whitney *U *test, *P* = 8.8 × 10^−^^7^) higher than secondary metabolic pathway genes (27.42 bp, *N* = 241). Both 5′- and 3′-UTR GC contents were significantly negatively correlated with *d*_N_, *d*_S_, and *d*_N_/*d*_S_ (Spearman’s rank correlation, *P* < 0.001) (table 2). Notably, we have also found that UTR GC content is significantly positively correlated with UTR length (Spearman’s ρ_5__′__-UTR length vs__.__ GC content_ = 0.371, *P* = 1.0 × 10^−^^6^ and Spearman’s ρ_3__′__-UTR length vs__.__ GC content_ = 0.608, *P* = 1.0 × 10^−^^6^). It is known that UTR regions are crucial for posttranscriptional regulation of gene expression (Mignone et al. 2002). In human, it was found that genes located in large GC-rich regions of a chromosome (heavy isochores) have shorter UTRs than genes located in GC-poor isochores (Mignone et al. 2002). Moreover, in vertebrates, it has been proposed that most housekeeping genes should be located in GC-rich isochores, whereas tissue-specific genes should be located in GC-poor isochores (Bernardi 2000). However, secondary metabolic pathway genes are more tissue specific and have shorter UTRs than primary metabolic pathway genes, as found in this study, which contradicts the situation in humans and vertebrates. Average GC_3_ content of primary metabolic pathway genes (0.412, *N* = 1,024) was significantly lower (Mann–Whitney *U *test, *P* = 3 × 10^−^^3^) than secondary metabolic pathway genes (0.423, *N* = 237). GC_3_ was significantly negatively correlated with *d*_N_ and *d*_N_/*d*_S_ but not *d*_S_ (table 2). It has been shown that recombination is a driving force for the increase in GC_3_ in many organisms (Tatarinova et al. 2010; Elhaik and Tatarinova 2012, p. 3). Although self-pollination in *Arabidopsis* keeps its recombination rates low, which ultimately results in reduced GC_3_ content, evolutionary pressure would selectively keep high recombination rates for some genes (Elhaik and Tatarinova 2012). After GC_3_ richness evolves in those genes under selective pressure, its additional transcriptional advantage is achieved (Elhaik and Tatarinova 2012). Moreover, Straussman et al. (2009) described a correlation between methylation and GC_3_. GC_3_-rich genes provide more targets for de novo methylation that can serve as an additional mechanism of transcriptional regulation that ultimately increases adaptability to a species under external stresses (Elhaik and Tatarinova 2012). The higher GC_3_ content of secondary metabolic genes in our study supports this view. As these genes provide a selective advantage to the plant under changing environmental conditions, the GC_3_ content became higher than primary metabolic pathway genes.

### Effect of Gene Expression

Average expression level of primary metabolic pathway genes (9,025.77, *N* = 1,004) was approximately 2.03 fold of secondary metabolic pathway genes (4,430.29, *N* = 235) and the difference was significant (Mann–Whitney *U *test, *P* = 1.79 × 10^−^^7^). The tissue specificity (τ = 0.287, *N* = 235) of secondary metabolic pathway genes was significantly (Mann–Whitney *U *test, *P* = 1.17 × 10^−^^12^) higher than primary metabolic pathway genes (τ = 0.236, *N* = 1,008). Both expression level and tissue specificity were significantly correlated with *d*_N_, *d*_S_, and *d*_N_/*d*_S_ (table 2). EN_c_ of primary metabolic pathway genes (53.18, *N* = 1,024) was also significantly lower (Mann–Whitney *U *test, *P* = 2.97 × 10^−^^7^) than secondary metabolic pathway genes (54.47, *N* = 237), indicating that primary metabolic pathway genes show more codon biasness than secondary metabolic pathway genes. We have also studied the relationship of expression level and tissue specificity with GC_3_ and EN_c_. Both GC_3_ and EN_c_ showed a significant correlation with expression level (Spearman’s ρ_expression level vs__.__ ENc_ = −0.184, *P* = 1.0 × 10^−^^6^; Spearman’s ρ_expression level vs__.__ GC3_ = 0.138, *P* = 3.3 × 10^−^^6^). GC_3_ and EN_c_ were significantly correlated with tissue specificity (Spearman’s ρ_τ vs__.__ ENc_ = −0.121, *P* = 4.6 × 10^−^^5^; Spearman’s ρ_τ vs__.__ GC3_ = 0.105, *P* = 3.81 × 10^−^^4^). EN_c_ was significantly positively correlated with *d*_N_ and *d*_N_/*d*_S_ but not *d*_S_ (table 2). There is a strong positive correlation between GC_3_ and EN_c_ (Spearman’s ρ_ENc vs__.__ GC3_ = 0.322, *P* = 1.0 × 10^−^^6^). It was reported that mRNA secondary-structure stability is correlated with both GC content and codon usage (Gu et al. 2010). Moreover, the common causes of heterologous gene expression are mainly associated with the disparities in codon bias, mRNA secondary structure and stability, gene product toxicity, and product solubility (Makrides 1996; Gustafsson et al. 2004). Hence, it is clear from this study that expressing secondary metabolic genes in another host such as bacteria or simple eukaryotes is easier than primary metabolic pathway genes. For heterologous expression of the latter, additional methods such as codon optimization (Angov 2011) or codon harmonization (Angov et al. 2008) may be required. For a better knowledge about the expression of genes with respect to different structures and developmental stages of the plant, we have also studied the PO terms (Avraham et al. 2008). The PO database uses 71 plant structure development stages and 326 plant anatomical entities to describe *Arabidopsis* gene expression patterns (Cooper et al. 2013). We have counted the number of PO terms per gene and correlated that with evolutionary rate. Primary metabolic pathway genes are involved in significantly more PO terms (28.25 per gene, *N* = 1,035; Mann–Whitney *U *test, *P* = 7.44 × 10^−^^12^) than secondary metabolic pathway genes (22.48 per gene, *N* = 241). There is a strong negative correlation between PO terms per gene and evolutionary rates (table 2).

### Multifunctionality

**Fig. 4.—:**
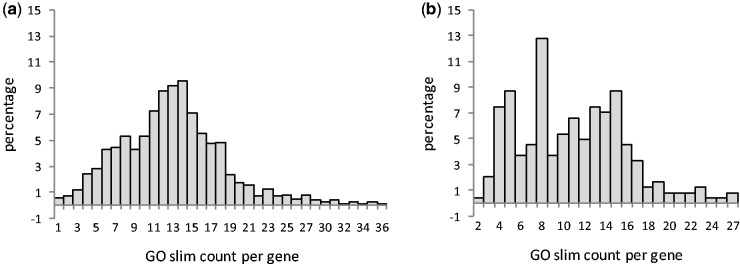
Distribution of GO slim numbers per gene in (*a*) primary and (*b*) secondary metabolic pathway genes in *A. thaliana*.

Primary metabolic pathway genes were found to be significantly more multifunctional (12.95 per gene, *N* = 1,035; Mann–Whitney *U *test, *P* = 1.72 × 10^−^^12^) than secondary metabolic pathway genes (10.91 per gene, *N* = 241) (fig. 4). There is a strong negative correlation between GO slim terms per gene and evolutionary rates (table 2). It shows that primary metabolic genes are evolutionarily more conserved due to their higher multifunctionality. It was also found that highest number (12.86%) of genes of the secondary metabolic pathways take part in eight biological processes whereas 9.56% genes of the primary metabolic pathways take part in 14 biological processes. Multifunctionality, that is, involvement of a protein in several processes has been shown by several enzymes such as hexokinase, triose-phosphate isomerase, enolase, etc. and the presence of multifunctional proteins increases the metabolic efficiency of a cell (Schwab 2003). This study shows that primary metabolic pathway genes are more multifunctional than genes involved in secondary metabolism. Hence, metabolic efficiency is primarily maintained by genes involved in primary metabolism. In yeast, it has been found that multifunctional proteins evolve at a slower rate (Salathé et al. 2006). It was also revealed in this study that multifunctionality has greater effect on *d*_N_ than *d*_S_. However, our analysis shows that the magnitude of the effect of multifunctionality on *d*_N_, *d*_S_, and *d*_N_/*d*_S_ of metabolic pathways is higher than its effect on whole genome of *A. thaliana *as studied by Yang and Gaut (2011). This shows that multifunctionality has greater effect on metabolic genes than other proteins of the genome. It is also notable that both expression level and tissue specificity are significantly correlated with multifunctionality (Spearman’s ρ_expression level vs__.__ multifunctionality_ = 0.547, *P* = 1.0 × 10^−^^6^; Spearman’s ρ_tissue specificity vs__.__ multifunctionality_ = −0.308, *P* = 1.0 × 10^−^^6^). In PCA, the first principal component includes expression parameters and multifunctionality. This contradicts with the result of Salathé et al. (2006) who have not found any significant correlation of multifunctionality with expression level. However, the reason behind the higher expression and lower tissue specificity of multifunctional genes of metabolic pathways is not clear yet. One possible explanation is that secondary metabolic pathway genes evolve in response to specific environmental factors and thus less multifunctional and more tissue specific. On the other hand, genes involved in primary metabolism are more multifunctional as this enhances metabolic efficiency. They are also ubiquitously expressed in the plant body at a higher level to successfully maintain the core functioning of the plant body. Number of domains per gene was significantly higher in genes involved in primary metabolic pathway genes than secondary metabolic pathway genes. It was also found that domain number is negatively correlated with evolutionary rate. The reason behind this correlation was not clear. Then, we correlated domain number with other parameters to investigate whether it is correlated with other factors. Domain number was significantly correlated with multifunctionality (Spearman’s ρ_domain numbers multifunctionality_ = 0.153, *P* = 1.0 × 10^−^^6^) as well as gene length (Spearman’s ρ_domain numbers gene length_ = 0.358, *P* = 1.0 × 10^−^^6^), intron length (Spearman’s ρ_domain numbers intron length_ = 0.188, *P* = 1.0 × 10^−^^6^), and GC_3_ (Spearman’s ρ_domain numbers GC3_ = −0.123, *P* = 7.0 × 10^−^^5^). In PCA, domain number along with gene length, intron number, and GC_3_ was included in principal component 2. Thus, it seems that domain number is more of a function of gene character rather than multifunctionality.

### Relative Involvement of the Factors in Shaping Evolutionary Rate Variation among Primary and Secondary Metabolic Pathway Genes

**Fig. 5.—:**
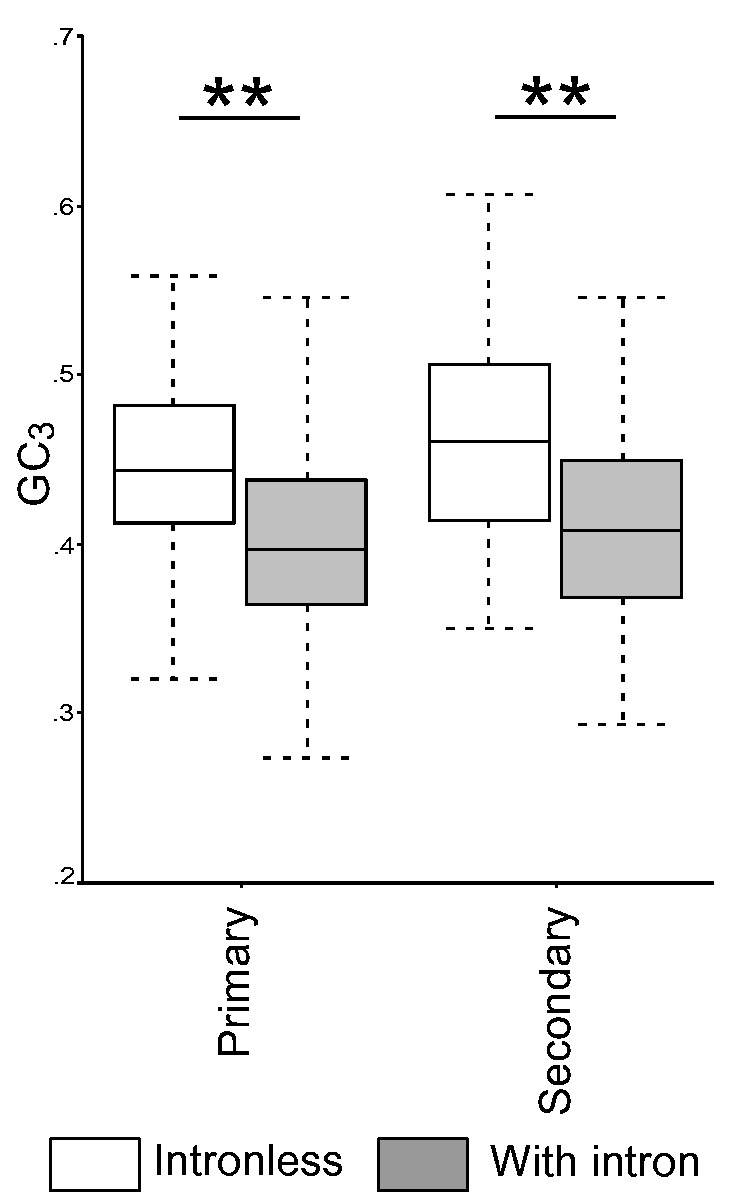
Comparison of GC_3_ content between intronless genes and genes with introns in primary and secondary metabolic pathway genes of *A. thalaiana*. The intronless genes showed significantly higher GC_3_ content than genes with intron (Mann–Whitney *U *test, **P < 0.01).

To elucidate the covariance structure of different factors, we have performed PCA. Results of PCA analysis are given in table 3. Components with values more than 0.5 have been retained. It has been observed that the first two principal components have explained 43.312% of the total variance. The major contributors of the first component were PO, 3′-UTR GC content, 5′-UTR GC content, expression level, tissue specificity, 3′-UTR length, and multifunctionality (table 3). The major contributors of the second principal component were gene length, intron number, GC_3_, and domain number. It is noteworthy that the first component showed higher correlation coefficient with *d*_N_ than *d*_S_ and the second component showed the reverse. The contributors of the first coefficient mainly comprised factors related to gene expression and the second coefficient mainly comprised gene-level factors. Hence, it is apparent that the difference in nonsynonymous substitutions in the two types of pathways is mainly due to factors related to gene expression, whereas the difference in synonymous substitutions is due to gene-level variations such as length, intron number, and domain number. However, the inclusion of GC_3_ content in the second component seems to be strange as GC_3_ has effect on the regulation of gene expression by methylation as discussed earlier. We have performed Spearman correlation of GC_3_ with gene length, intron number, and domain number. Indeed, GC_3_ content is significantly negatively correlated with intron number (Spearman’s ρ = −0.499, *P* = 1.0 × 10^−^^6^), gene length (Spearman’s ρ = −0.326, *P* = 1.0 × 10^−^^6^), and domain number (Spearman’s ρ = −0.122, *P* = 7.1 × 10^−^^5^). Moreover, it has been found in corn that genes with high GC_3_ tend to be mono-exonic (Alexandrov et al. 2009). We have also found a similar trend in both primary and secondary metabolic pathway genes in *A. thaliana* (fig. 5). Thus, it is clear that intron number is highly correlated with GC_3_ content. Probably, the correlation is due to the accumulation of more synonymous mutations in intronic regions, which also explains the significant negative correlation with gene length. As genes of the primary metabolic pathways are longer than secondary metabolic pathway genes and their coding sequence length is not significantly different, it is, thus, evident that the increased gene length in primary metabolic genes is mainly contributed by introns, and thus gene length shows a significant negative correlation with GC_3_. However, the correlation between GC_3_ and domain number is not very clear. The *d*_N_/*d*_S_ ratio showed higher correlation coefficient with component 1 than component 2. Thus, it is clear that the evolutionary rate difference between primary and secondary metabolic pathway genes is chiefly due to the factors related to expression than gene-level predictors in *A. thaliana*.

**Table 3:** PCA on *d*_N_, *d*_S_, and *d*_N_/*d*_S_ and Major Contributors of the Principal Components

	Principal Component 1	Principal Component 2
Percent of the total variance	26.164	17.148
Correlation coefficient (Spearman’s ρ) with *d*_N_	−0.439 (*P* = 1.0 × 10^−6^)	−0.013 (*P* = 6.8 × 10^−1^)
Correlation coefficient (Spearman’s ρ) with *d*_S_	−0.207 (*P* = 1.0 × 10^−6^)	−0.158 (*P* = 1.0 × 10^−6^)
Correlation coefficient (Spearman’s ρ) with *d*_N_/*d*_S_	−0.361 (*P* = 1.0 × 10^−6^)	−0.044 (*P* = 1.5 × 10^−1^)
Major contributors		
PO	0.740	
3′-UTR GC content	0.754	
5′-UTR GC content	0.689	
3′-UTR length	0.586	
GO slim	0.581	
Tissue specificity	−0.660	
Expression level	0.533	
Gene length		0.869
Intron number		0.857
GC_3_		−0.616
Domain number		0.567

## Conclusion

This study showed that primary metabolic pathway genes are evolutionary more conserved than secondary metabolic pathway genes in *A. thaliana*. The effect of different gene level expression level and protein level factors showed that gene length, gene compactness, expression level, tissue specificity, multifunctionality, and domain number are the major contributors for the evolutionary rate difference of these primary and secondary metabolic pathway genes. To the best of our knowledge, this is the first extensive comparison of primary and secondary metabolic pathway genes from an evolutionary perspective. Improving the agronomic quality of a crop by altering its metabolic signature by targeted breeding can be an important tool for a breeder. The knowledge gathered from this study can play a pivotal role for this kind of breeding practices. As secondary metabolic pathway genes are less conserved, their intra- or interspecific variation should be greater, and this variation can be a starting point for transgenic manipulation or targeted breeding. Moreover, as these genes tend to accumulate more substitutions, protein engineering by site-directed mutagenesis can lead to the formation of a plethora of new economically important metabolites. On the other hand, when targeting a primary metabolic gene, emphasis should not be to alter its coding sequence as this can disrupt the function of these highly conserved genes that will ultimately affect the plant phenotype. Rather, for improved production of primary metabolites, factors that affect gene expression such as UTRs or other regulatory elements may be altered. Alternately, for successful heterologous expression, codon optimization or codon harmonization may be beneficial. Our study, thus, provides valuable information on the evolutionary aspects of primary and secondary metabolism in *A. thaliana* which, along with further laboratory-based experimental studies, can be helpful for metabolic engineering and production of improved plant varieties in the near future.

## Supplementary Material

Supplementary materials S1 and S2 are available at *Genome Biology and Evolution *online (http://www.gbe.oxfordjournals.org/).

Supplementary Data
