# Supplementary material for: Evolutionary Rate Heterogeneity of Primary and Secondary Metabolic Pathway Genes in Arabidopsis thaliana
Source: Genome Biol Evol. 2015 Nov 10;8(1):17–28. doi: 10.1093/gbe/evv217 (PMC4758233; doi:10.1093/gbe/evv217)
Supplement: Supplementary Data [file supp_8_1_17__index.html]

Evolutionary rate heterogeneity of primary and secondary metabolic pathway genes in Arabidopsis thaliana — Evolutionary Rate Heterogeneity of Primary and Secondary Metabolic Pathway Genes in Arabidopsis thaliana — Supplementary Data 

# Evolutionary Rate Heterogeneity of Primary and Secondary Metabolic Pathway Genes in *Arabidopsis thaliana*

## Supplementary Data

files

- Supplementary Data - xlsx file
- Supplementary Data - doc file
