# Supplementary material for: Evolutionary Rate Heterogeneity of Primary and Secondary Metabolic Pathway Genes in Arabidopsis thaliana
Source: Genome Biol Evol. 2015 Nov 10;8(1):17–28. doi: 10.1093/gbe/evv217 (PMC4758233; doi:10.1093/gbe/evv217)
Supplement: Supplementary Data [file supp_evv217_Supplementary_material_S1.doc]

Supplementary material S1: List of all primary and secondary metabolic pathways of *Arabidopsis thaliana* studied.

**Primary metabolic pathways**

Carbohydrate metabolism: Glycolysis / Gluconeogenesis, Pentose phosphate pathway, Pentose and glucuronate interconversions, Fructose and mannose metabolism, Galactose metabolism, Ascorbate and aldarate metabolism, Starch and sucrose metabolism, Amino sugar and nucleotide sugar metabolism, Pyruvate metabolism, Glyoxylate and dicarboxylate metabolism, Propanoate metabolism, Butanoate metabolism, Branched dibasic acid metabolism, Inositol phosphate metabolism,

Energy: Oxidative phosphorylation, Photosynthesis, Photosynthesis - antenna proteins, Carbon fixation in photosynthetic organisms, Nitrogen metabolism, Sulfur metabolism

Lipid metabolism: Fatty acid biosynthesis, Fatty acid elongation, Fatty acid degradation, Synthesis and degradation of ketone bodies, Cutin, suberine and wax biosynthesis, Steroid biosynthesis, Glycerolipid metabolism, Glycerophospholipid metabolism, Ether lipid metabolism, Sphingolipid metabolism, Arachidonic acid metabolism, Linoleic acid metabolism, alpha-Linolenic acid metabolism, Biosynthesis of unsaturated fatty acids,

Nucleotide metabolism: Purine metabolism, Pyrimidine metabolism,

Amino acid metabolism: Alanine, aspartate and glutamate metabolism, Glycine, serine and threonine metabolism, Cysteine and methionine metabolism, Valine, leucine and isoleucine degradation, Lysine biosynthesis, Arginine and proline metabolism, Histidine metabolism, Tyrosine metabolism, Phenylalanine metabolism, Tryptophan metabolism,

Metabolism of other amino acids: beta-Alanine metabolism, Taurine and hypotaurine metabolism, Selenocompound metabolism, Cyanoamino acid metabolism, D-Glutamine and D-glutamate metabolism, Glutathione metabolism

Glycan biosynthesis and metabolism: N-Glycan biosynthesis, O-glycan biosynthesis, Glycosaminoglycan degradation, Glycosylphosphatidylinositol(GPI)-anchor biosynthesis, Glycosphingolipid biosynthesis-- globo series, Glycosphingolipid biosynthesis - ganglio series, Other glycan degradation

Metabolism of cofactors and vitamins: Thiamine metabolism, Riboflavin metabolism, Vitamin B6 metabolism,

**Secondary metabolic pathways**

Metabolism of terpenoids and polyketides: Terpenoid backbone biosynthesis, Monoterpenoid biosynthesis, Sesquiterpenoid and triterpenoid biosynthesis, Diterpenoid biosynthesis, Carotenoid biosynthesis, Brassinosteroid biosynthesis, Zeatin biosynthesis, Limonene and pinene degradation, Polyketide biosynthesis proteins, Biosynthesis of siderophore group nonribosomal peptides

Biosynthesis of other secondary metabolites: Phenylpropanoid biosynthesis, Stilbenoid, diarylheptanoid and gingerol biosynthesis, Flavonoid biosynthesis, Anthocyanin biosynthesis, Indole alkaloid biosynthesis, Isoquinoline alkaloid biosynthesis, Tropane, piperidine and pyridine alkaloid biosynthesis, Caffeine metabolism, Betalain biosynthesis, Glucosinolate biosynthesis, Butirosin and neomycin biosynthesis, Aflatoxin biosynthesis
